# Seasonal Changes in the Character and Nitrogen Content of Dissolved Organic Matter in an Alpine/Subalpine Headwater Catchment

**DOI:** 10.1100/tsw.2001.273

**Published:** 2001-10-30

**Authors:** Eran W. Hood, Mark W. Williams

**Affiliations:** Departmetn of Geography and Institute for Arctic and Alpine REsearch, University of Colorado, Boulder 80309-0450, USA

**Keywords:** DON, DOC, nutrient cycling, alpine hydrology, Colorado Front Range, N deposition

## Abstract

We are studying the chemical quality of dissolved organic nitrogen (DON) in a high-elevation watershed in the Colorado Front Range. Samples were collected over the 2000 snowmelt runoff season at two sites across an alpine/subalpine ecotone to understand how the transition between the lightly vegetated alpine and forested reaches of the catchment influences the chemical character of DON. Samples were analyzed approximately weekly for dissolved organic material (DOM) content and chemical character. A subset of samples was analyzed for the elemental content of fulvic and hydrophilic acids. Concentrations of DON at both sites were highest in the spring at the initiation of snowmelt, decreased during snowmelt, and increased again during the late summer and fall. In contrast, concentrations of dissolved organic carbon (DOC) peaked on the ascending limb of the hydrograph and declined to seasonal minima on the descending limb of the hydrograph. The ratio of DOC:DON showed a seasonal shift at both sites with high values (40 to 55) during peak runoff in early summer and lower values (15 to 25) during low flows late in the runoff season. These results indicate that there was a seasonal change in the relative N content of DOM at both sites. Chemical fractionation of DOC showed that there were temporal and longitudinal changes in the chemical character of DOC. At the alpine site, the fulvic acid content of DOC decreased from 57% in June to 35% in September. The change in fulvic acid was less pronounced at the forested site, from 66% in June to 54% in September. Elemental analysis of fulvic and hydrophilic acids indicated that hydrophilic acids were N rich compared to fulvic acids. Additionally, fulvic and hydrophilic acids isolated at the alpine site had a lower C:N ratio than those isolated at the forested site. Similarly, the C:N ratio of organic acids at both sites was lower in September than in June during peak runoff. These differences appear to be a result of changes in both DOM precursor material and hydrologic flowpaths. Using C:N ratios of fulvic and hydrophilic acids, we estimate that nonhumic material carried between 54 to 73% of the organic N in surface water at the alpine site and 44 to 58% of the organic N in surface water at the subalpine site.

## INTRODUCTION

The global nitrogen (N) cycle is undergoing changes as a result of human activities[1,2]. At present, dissolved organic nitrogen (DON) is the dominant form of N in surface waters of many forested[3,4,5] and high-elevation alpine environments[6,7]. Additionally, DON has been shown to be an important source of nutrients for plants[8,9] and aquatic bacteria[10]. Experimentally increased levels of inorganic N deposition do not appear to alter DON exports to the same extent as inorganic N exports[11,12]. However, it is possible that N deposition may alter soil biogeochemical processes, which could, in turn, alter the chemical character of DON leached to surface waters.

The chemical character of DON has been studied primarily in soil solutions[13], and little is known about the composition of DON found in surface waters. In the high-elevation North Boulder Creek catchment in the Colorado Front Range, Williams et al.[14] found that the C:N ratio of dissolved organic matter (DOM) decreased after peak snowmelt, indicating that the proportion of N in DOM increases late in the runoff season. In a similar high-elevation system, McKnight et al.[15] found that aquatic fulvic acids in the subalpine reach of the Loch Vale catchment in Rocky Mountain National Park had a high N content relative to the N content reported for fulvic acids at other forested sites. These findings suggest that DON cycling has the potential to be influenced by changes in the N content of terrestrial and aquatic DOM precursor material resulting from increased deposition of inorganic N. Fractionation of DOM into operationally defined chemical constituents has shown that nonhumic material typically has a higher N content than fulvic acids, which comprise the majority of humic DOM in surface waters[16]. Thus, altering the balance of humic and nonhumic material has the potential to alter both the quantity and quality of aquatic DON. However, little work has been done to identify the DOM fractions with which DON is associated in surface waters, particularly at the watershed scale.

Headwater streams transport water and dissolved nutrients from terrestrial to downstream aquatic systems and play a disproportionately large role in N transformations within ecosystems[17]. Here we report on measurements of DON from a high-elevation headwater stream in the Colorado Front Range of the Rocky Mountains. We also include measurements of dissolved organic carbon (DOC) in order to investigate changes in the C:N ratio of organic material in surface waters. We employ chemical fractionation and elemental analysis in order to elucidate the character of DON in surface waters. To better understand the controls on the character of DON, we evaluate seasonal and longitudinal changes in both the proportion and the C:N ratio of humic and nonhumic material in the aquatic DOM load.

## SITE DESCRIPTION

Green Lakes Valley is an alpine/subalpine watershed containing seven lakes connected by North Boulder Creek (Fig. 1). Above Lake Albion, the watershed is alpine in nature, dominated by exposed bedrock and talus fields, with patches of alpine tundra on ridge tops and in the valley bottom. Soils are a mixture of Cryic Inceptisols and Entisols, and are limited in extent, depth, and development as a result of late-Pleistocene glaciation[18]. Treeline is located just below Lake Albion. Below this point the watershed is forested and composed primarily of subalpine fir (*Abies lasiocarpa*) and Engelmann spruce (*Picea engelmannii)* with some limber pine (*Pinus flexilis*) at treeline and regrowth lodgepole pine (*P. contorta*) lower in the basin. The subalpine portion of the catchment is typified by developed soils on glacial till and moraine deposits overlying granitic and metamorphic parent material. Soils are Inceptisols and intermixed Alfisols with Histosols in wet meadow areas.

Approximately 80% of the annual precipitation occurs as snow that typically accumulates from October to April[19]. Inorganic N deposition in wetfall is approximately 4 kg $ha^{-1}$ $year^{-1}$[20]. Snowmelt is the dominant hydrologic event of the year, and streamflow is markedly seasonal varying from less than 0.1 $m^3 s^{-1}$ during the winter months to greater than 1.5 $m^3 s^{-1}$ during spring snowmelt just below Lake Albion near treeline (Fig. 1). For this study, samples of organic material were collected at three locations: the outlet of Green Lake 4 (GL4), the outlet of Green Lake 3 (GL3), and the City of Boulder gauge (SLP) 2 km below Silverlake Reservoir (Fig. 1).

## METHODS

### Sampling and Analytical Techniques

During the 2000 snowmelt season (April to November), surface water samples were collected as 1-L grab samples at weekly to biweekly intervals at the GL4 and SLP sites. Samples were collected in precombusted amber glass bottles with Teflon-lined caps and filtered through precombusted Gelman G/F glass fiber filters with a nominal pore size of 0.7 µm. The majority of samples were filtered in the field, and all samples were filtered within a day of collection. Samples were stored at 4°C until fractionation and analysis, which occurred within 2 weeks of sample collection.

A 200-ml subsample of every 1-L surface water sample was fractionated into hydrophobic (humic) and hydrophilic (nonhumic) fractions using analytical scale column chromatography with XAD-8 Amberlite resin according to the methods of Thurman and Malcolm[21]. The hydrophobic fraction, which sorbs to the XAD-8 resin, is composed of fulvic and humic acids. In the surface waters we investigated, the hydrophobic fraction is thought to be composed primarily of fulvic acids (typically >90%)[22], therefore we refer to the hydrophobic fraction as fulvic acids following the terminology of Baron et al.[23]. The fulvic acid fraction was determined by back-eluting the XAD-8 resin with 0.1 N NaOH. The DOC concentration of the eluate was measured after acidification to pH 2 with concentrated phosphoric acid. We define the nonhumic fraction as a heterogeneous class of substances that pass through the XAD-8 resin. The nonhumic hydrophilic fraction is composed predominantly of hydrophilic organic acids and low molecular weight compounds including carbohydrates, carboxylic acids, and amino acids[22]. The nonhumic fraction was calculated by measuring the DOC concentration of the effluent from the XAD-8 resin. Based on fractionation of replicate samples, the precision of our fractionation procedure was 5 to 10%.

All water samples were also analyzed for N species at the Kiowa Environmental Chemistry Laboratory, located approximately 6 km from North Boulder Creek. Nitrate ($NO_3$-N) was determined using a Dionex DX 500 ion chromatograph with an IonPac AS4A-SC Analytical Column. Ammonium ($NH_4$-N) was determined on a Lachat QuikChem 4000 Flow Injection Analyzer using a phenolate reaction enhanced by nitroprusside.

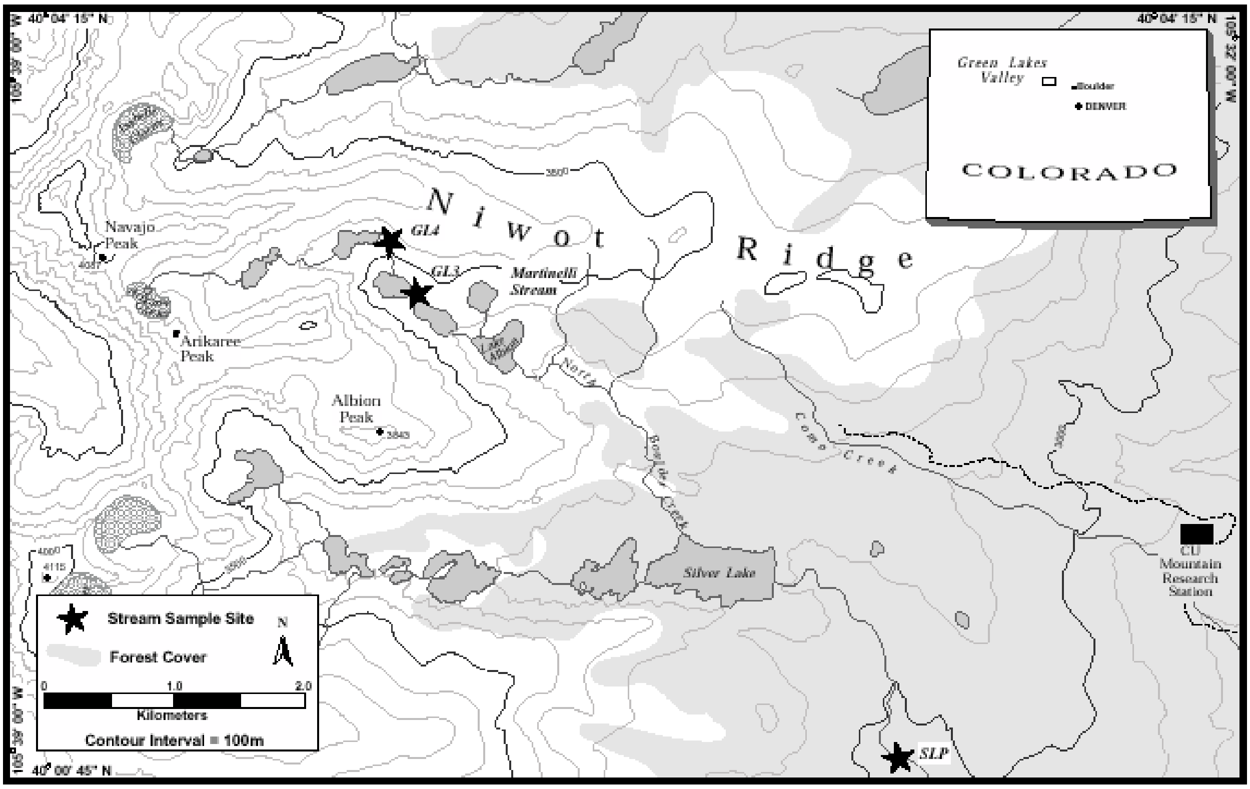

**FIGURE 1**. Topographic map of the North Boulder Creek catchment in the Green Lakes Valley. North Boulder Creek originates from the Arikaree glacier, just east of the Continental Divide (3900 m.a.s.l.). Stream sampling sites for 2000 are denoted by stars. GL4 and GL3 are located in the alpine reach of the catchment. SLP is located in the subalpine forest, 5 km below treeline.

Total nitrogen (TN) was determined using an Antek 9000N TN analyzer. DON was calculated by subtracting measured inorganic N ($NO_3$-N + $NH_4$-N) from the TN value of the sample. DOC was determined by high-temperature catalytic oxidation using a Shimadzu Organic Carbon Analyzer at the Institute of Arctic and Alpine Research in Boulder, CO.

Large volume (100 L) samples for isolating preparative quantities of fulvic acid (FA) and hydrophilic acid (HPIA) were collected at the GL3 and SLP sites on June 8 and September 14, 2000. The GL3 site was sampled because it was not possible to transport 100 L of water from GL4. However, previous surface water sampling and discharge measurements have shown that the GL3 and GL4 sites are very similar in terms of discharge and water chemistry[24]. We followed the fractionation procedure of Aiken et al.[16] to isolate fulvic acids and hydrophilic acids. Columns (2 l) of XAD-8 and XAD-4 resin were connected in series by Teflon tubing. Samples were filtered through a Balston glass fiber filter type AAH (0.3µ) and acidified to pH 2. Approximately 100 L of sample was passed through the XAD-8/XAD-4 column pair. After rinsing with deionized water, the XAD-8 column was back-eluted with 4 L of 0.1 NaOH to isolate the fulvic acid fraction and the XAD-4 column was back eluted with 4 L of 0.1 NaOH to isolate the hydrophilic acid fraction. The eluents from both columns were hydrogen-saturated using a cation exchange resin and then freeze-dried within 2 days. Lyophilized fulvic acid and hydrophilic acid samples were analyzed at Huffman Laboratories in Golden, Colorado for C and N content using individual elemental analyzers according to the procedures of Huffman and Stuber[25].

## Calculation of Fulvic and Nonhumic N

Attempts to measure fractionated DON directly were unsuccessful because the acid matrix of the DOM fractions interfered with the measurement of total dissolved N. As a result, the percentage of DON associated with the fulvic acid and nonhumic material was determined in the following manner: Concentrations of DOC associated with the fulvic acid and nonhumic fractions were calculated at GL4 and SLP on June 8 and September 14 using the results from the analytical scale column fractionation. The C:N ratios of fulvic and hydrophilic acid isolated on the same date were then used to calculate DON concentrations from the DOC concentrations (here we assume that the difference in the C:N ratio of organic material does not change appreciably between GL4 and GL3). It is important to note that the C:N ratio of hydrophilic acid was used to calculate the DON concentration for the entire nonhumic fraction. This assumption likely produces a small underestimate of the DON concentration associated with the nonhumic fraction because, in addition to hydrophilic acids, the nonhumic fraction contains small amounts of low molecular weight compounds, including carbohydrates, carboxylic acids, and amino acids that can be relatively N-rich[13].

## RESULTS AND DISCUSSION

### Hydrology

The hydrograph at the GL4 site from May to October 2000 demonstrated the markedly seasonal flows characteristic of snowmelt dominated catchments. Daily streamwater discharge began a rapid, steady increase the week of May 20 and peaked at greater than 27,000 $m^3$ $day^{-1}$ on May 30 coincident with the breaching of an ice dam just upstream in GL5 (Fig. 2). Discharge at GL4 then decreased rapidly but remained between 15,000 to 20,000 $m^3$ $day^{-1}$ for much of June and early July.

### Concentrations of DON and DOC

There were clear seasonal patterns in concentrations of DON and DOC at both sample sites (Fig. 3, A and B). At the alpine GL4 site, concentrations of DON were 3 to 15 µmoles $L^{-1}$ in the spring before the initiation of snowmelt, decreased to <5 µmoles $L^{-1}$ during snowmelt, and increased again during the late summer and fall. The pattern for DON was similar at the forested SLP site, although concentrations were higher in the spring and late summer/fall periods and decreased below 5 µmoles $L^{-1}$ on only two sample dates during peak snowmelt runoff. The increase in DON concentrations in the fall is consistent with previous research on surface waters in the Lake Tahoe basin[7]. A similar trend in soil solution at the Harvard Forest was attributed to hydrologic flushing of accumulated decomposition products[11]; however, this mechanism is unlikely to be important for DON production in the alpine reaches of our study catchment which is dominated by exposed bedrock and talus.

Concentrations of DOC peaked on the ascending limb of the hydrograph in late May, consistent with the flushing of soluble organic carbon from near-surface soil horizons during the initiation of snowmelt runoff[26,27]. For the season, concentrations of DOC were typically about three times higher at the subalpine SLP site compared to the alpine GL4 site. Concentrations of DOC declined to seasonal minima at both GL4 (<1 mg C $L^{-1}$) and SLP (~2 mg C $L^{-1}$) during late season low flows. The late season increase in DON concentrations evident at the two sites was not matched by an increase in DOC. This was reflected in the seasonal change in the ratio of DOC:DON. At GL4, the DOC:DON ratio was above 40 in May and June and less than 15 late in the season, which was similar to previous results of Williams et al.[14] at the GL4 site. The DOC:DON ratio at SLP was about 50 early in the season and declined to 20 to 25 in the fall. These ranges in the DOC:DON ratio are consistent with the results of Curtis and Adams[28], who report DOC:DON ratios of 9 to 48 from a survey of previous studies on streams and rivers.

### Chemical Character of DOM

Chemical fractionation results from GL4 and SLP during peak snowmelt runoff (June 8, Fig. 2) and late season low flows (September 14, Fig. 2) indicate that there was both a seasonal and longitudinal change in the balance of fulvic acid vs. nonhumic materials in surface waters. During peak snowmelt, DOM at the subalpine SLP site had a fulvic acid content of 66% (Fig. 4). This is on the high end for natural waters, which typically consist of 30 to 50% humic substances[22]. The proportion of fulvic acid was lower at the GL4 site in June, indicating that humic substances comprise a lower percentage of the DOM load in the lightly vegetated upper reaches of the catchment. At both sites, there was a pronounced shift in the composition of DOM in the samples collected during low streamflow in September. The fulvic acid content of DOM decreased at both sites, to 54% at SLP and 35% at GL4 (Fig. 4). This seasonal shift in the chemical character of DOM is consistent with previous work in alpine/subalpine catchments[15,23,29]. The implication of this finding is that there is a shift toward DOM precursor material that contains less humic material on the descending limb of the snowmelt hydrograph.

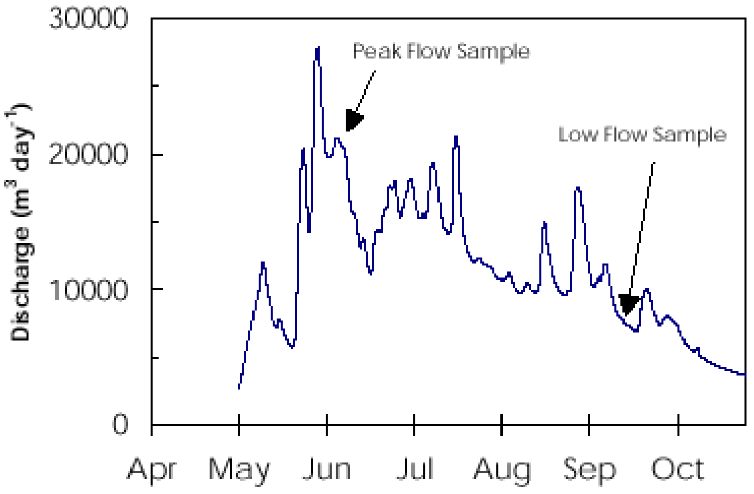

**FIGURE 2**. Daily discharge at the GL4 site for May to October 2000. Collection dates (June 8 and September 14, 2001) for the large volume samples used to isolate preparative quantities of organic material are denoted by arrows. Preparative quantities of fulvic acids and hydrophilic acids were used for elemental C and N analysis.

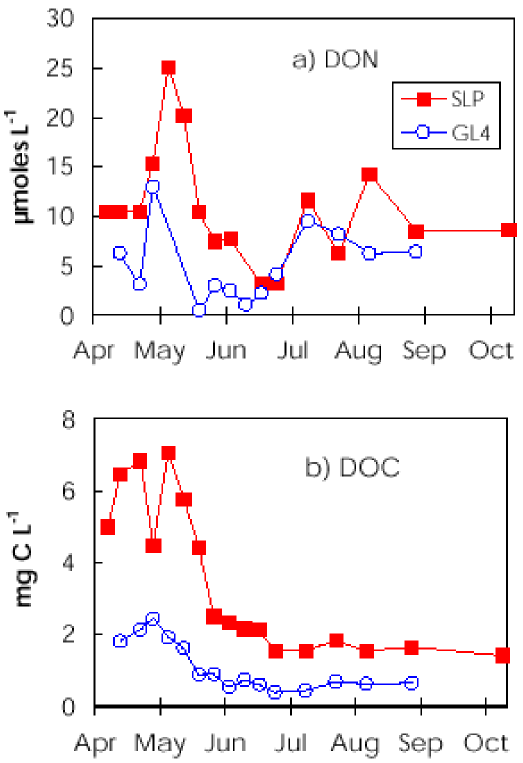

**FIGURE 3**. Concentrations of DON (A) and DOC (B) in streamwater at the alpine GL4 and subalpine SLP sites during May to October 2000. GL4 is denoted by open circles and SLP is denoted by squares. DON concentrations are in µmoles $L^{-1}$ and DOC concentrations are in mg C $L^{-1}$. Concentrations of both DON and DOC were higher at SLP although the seasonal pattern was similar at both sites.

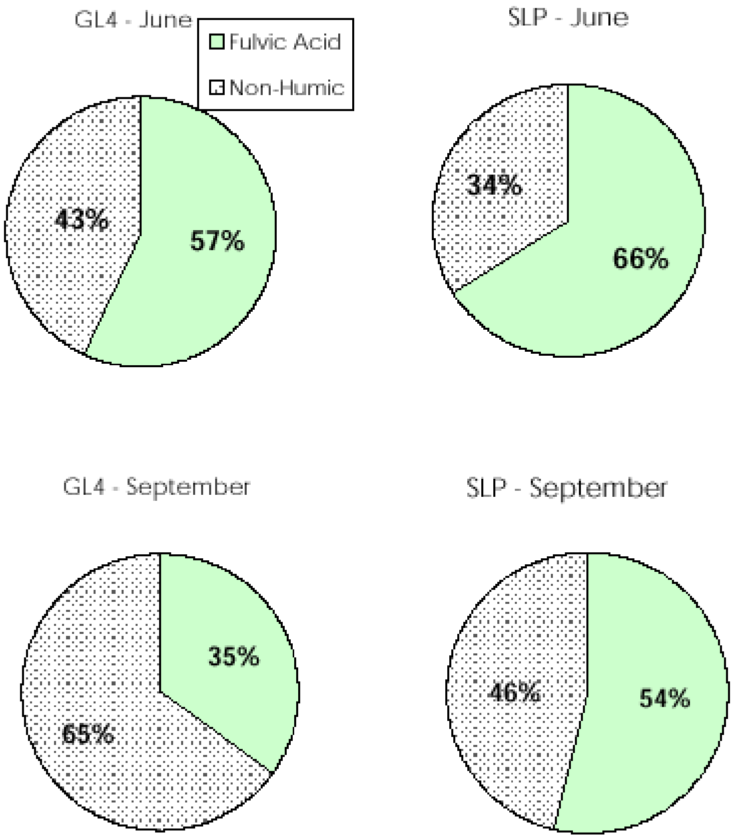

**FIGURE 4**. Proportion of DOC associated with fulvic acids and nonhumic material at GL4 and SLP during early season high flows (June 8) and late season low flow (September 14). Fulvic acids were the dominant carrier of organic C on both dates at SLP. At GL4, the dominant portion of organic C switched from fulvic acids to nonhumic material on the later sampling date.

Alternatively, the observed shift in chemical character of DOM at the two sites could be a result of changes in the hydrologic flowpath by which snowmelt enters the stream channel. Both sorption and biodegradation in mineral soil horizons have been shown to exert a strong influence on the chemical composition of DOM[13,30]. Thus, a seasonal change in the amount of water contacting deeper soil horizons could change the quality of DOM in the stream. The increase in the DOC:DON ratio of streamwater appears to be due to an increase in the ratio of nonhumic material to fulvic acids in September.

## Elemental Content of DOM Fractions

The C:N ratio of fulvic and hydrophilic acids demonstrated consistent temporal and longitudinal patterns within the catchment. The C:N ratios of the 4 fulvic acid isolates were 52 to 59% higher than the C:N ratios of the hydrophilic acid isolates (Table 1), consistent with the observation that humic material typically has a low N content relative to the N content of hydrophilic acids[16]. Interestingly, the fulvic and hydrophilic acid fractions collected at the alpine site had lower C:N ratios than the corresponding subalpine DOM fractions on both sample dates. The difference in N content was more pronounced during June, when C:N values were nearly 100% greater at SLP compared to GL3. This suggests that the soluble organic matter flushed into the stream in the subalpine is derived from terrestrial plant material, which tends to have a high C:N ratio, whereas the DOM in the alpine is derived from precursor material, which is relatively richer in N. One likely source of N-rich organic material is DOM derived from algal production in alpine lakes and lake sediments[23,31]. Previous research in high-elevation catchments in Colorado has linked DOC production to overwinter microbial activity[32]. Thus, the lower C:N ratios in the alpine may also reflect the fact that inorganic N inputs to the terrestrial watershed are relatively high[33] and microbial activity in the talus fields which mantle much of the upper basin appears to be C-limited[34]. Moreover, results from the Harvard Forest indicate that DOC fluxes are not related to inorganic N loading and ratios of DOC:DON often decrease with increased N inputs[11]. McKnight et al.[15] have previously hypothesized that the low C:N ratios of fulvic acids in the alpine/subalpine Loch Vale catchment in nearby Rocky Mountain National Park may be a result of sustained high deposition of N. With the exception of the subalpine fulvic acid collected on June 8, the C:N ratios we report for fulvic acids are similar to or lower than those reported by McKnight et al. Therefore it is possible that N deposition in high-elevation areas of the Colorado Front Range may be influencing the N content of DOM in our study catchment.

On a seasonal basis, the fulvic and hydrophilic acid samples collected at both sites during low flows in the fall had lower C:N ratios compared to samples collected during peak runoff (Table 1). This finding is important because it indicates that there is a seasonal change in both the chemical composition of DOM (the ratio of fulvic acid to nonhumic material), and in the relative C and N content of the aquatic fulvic and hydrophilic acids. At GL3, the C:N ratio of both the fulvic and hydrophilic acids fractions decreased by 31% between May and September. At SLP, the difference was even more pronounced with an approximately 50% decrease in the C:N ratio over the same time period. These findings suggest that the change in the elemental content of the major fractions of aquatic DOM may be more important than the shift in the balance of humic and nonhumic material in determining the N content of aquatic DOM. Overall, the lower C:N ratios of fulvic and hydrophilic acids at GL3 and SLP in the fall are consistent with the increase in DON concentrations relative to DOC concentrations evident at the two sites late in the season.

**TABLE 1**
**Atomic C:N Ratios of for Fulvic Acids (FA) and Hydrophilic Acids (HPIA) Collected at GL3 and SLP near Peak Snowmelt Runoff (June 8) and during Low Flows in the Fall (September 14)**

| | GL3 | | SLP | |
|---|---|---|---|---|
| | FA | HPIA | FA | HPIA |
| June | 27.8 | 18.3 | 52.8 | 34.3 |
| September | 21.2 | 13.9 | 35.2 | 22.1 |

## Character of DON

Unlike DOC, DON was associated primarily with the nonhumic fraction at both sites. This was particularly true at GL4 where the proportion of nonhumic DON was 54% at peak runoff and increased to greater than 70% late in the season (Fig. 5). At SLP, there was a shift from a predominance of humic DON in June (56%) to nonhumic DON in September (58%) (Fig. 5). These findings are consistent with Qualls and Haines[13] who found that hydrophilic acids carried the majority of DON in surface waters at the Coweeta Hydrologic Laboratory in North Carolina despite the fact that hydrophobic acids were the main DOC fraction. In our study, the high N content of hydrophilic acids suggests that they are a more important carrier for N compared to fulvic acids (Table 1). Moreover, the seasonal increase in the importance of hydrophilic acids as an N carrier at both sites is consistent with seasonal changes in flowpath that occur in snowmelt dominated catchments. During late season low flows, as much as 80 to 90% of the water entering the stream may be routed through mineral soil horizons[35]. Consequently, the predominance of hydrophilic acids is likely a result of a decrease in hydrophobic acids with depth in the soil profile due to preferential sorption of this fraction[13,36].

On both the June 8 and September 14 sampling dates, the percentage of DON associated with nonhumic material was higher at the alpine site than the subalpine site. This finding again reflects differences in DOM precursor material between these two reaches of the catchment. Previous research at GL4 has shown that instream production is responsible for nearly half of the DOM yield in the alpine zone[29]. DOM derived from algal precursor material has a relatively low fulvic acid content and is rich in N compared to DOM derived from terrestrial precursor material[31]. Therefore, it is not surprising that the majority of DON at the alpine site is carried in the nonhumic fraction of DOM. The relatively high fraction of DON associated with humic material at the subalpine site is consistent with organic material derived from humified terrestrial sources, particularly early in the snowmelt runoff season.

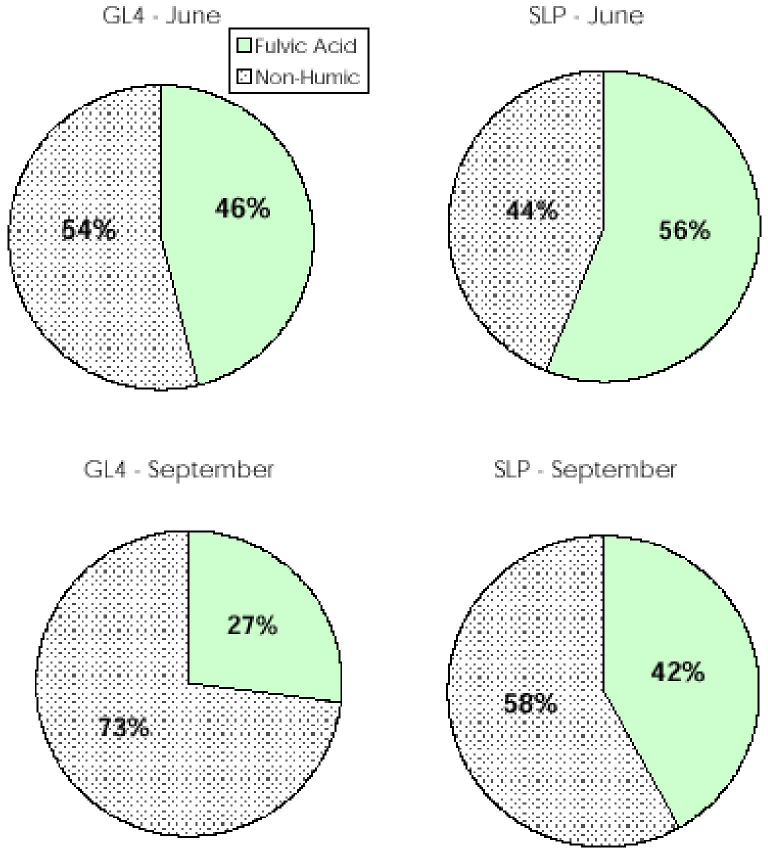

**FIGURE 5**. Proportion of DON associated with fulvic acids and nonhumic material at GL4 and SLP during early season high flows (June 8) and late season low flow (September 14). Unlike DOC, the nonhumic fraction was the dominant carrier of organic N for both sample dates at GL4 and for the late season sample date at SLP.

## CONCLUSIONS

There were substantial temporal and longitudinal changes in the chemical character of DON in the North Boulder Creek catchment over the snowmelt runoff season. Our results supported the notion that changes in the relative balance of humic and nonhumic material has important implications for DON quality in surface waters. Additionally, it appeared that the elemental composition of both fulvic and hydrophilic acids changes on relatively short temporal and spatial scales. The differences in the physical characteristics between the alpine and subalpine reaches of the catchment provided the opportunity to make an initial assessment of the ecological controls on the chemical character of DON. Our findings suggested that both biogeochemical parameters (such as landcover type, primary productivity, and microbial activity) and hydrologic parameters (such as flowpaths to the stream channel) play an important role in determining the quality of DON in surface waters. These insights should prove useful for understanding the controls on DON quality in other ecosystems that are being altered by atmospheric deposition of inorganic N.

## ACKNOWLEDGMENTS

James Weishaar and George Aiken provided advice on fractionation techniques and the use of their lab facilities at the U.S. Geological Service office in Boulder, CO. Tom Davinroy provided the map for Fig. 1. Kurt Chowanski helped with field work. Chris Seibold and the staff of the Kiowa Lab provided inorganic N analyses. Nel Caine provided streamflow data. An earlier version of the manuscript was improved substantially by comments from Paul Brooks, John Campbell, and James Sickman. Support for this research came from the National Park Service Air Resources Division and National Science Foundation grants DEB 0087248 and DEB 9810218 to the NWT LTER. EWH received additional support from a graduate research traineeship (GRT) grant in hydrology from the National Science Foundation.

## References

1. Galloway, J.N., Schlesinger, W.H., Levy, H., Michaels, A., and Schnoor, J.L. (1995) Nitrogen fixation: anthropogenic enhancement-environmental response. *Glob. Biogeochem. Cy.* **9(2),** 235–252.
2. Vitousek, P.M., Aber, J.D., Howarth, R.W., Likens, G.E., Matson, P.A., Schindler, D.W., Schlesinger, W.H., and Tilman, D.G. (1997) Human alteration of the global nitrogen cycle: sources and consequences. *Ecol. Appl.* **7,** 737–750.
3. Hedin, L.O., Armesto, J.J., and Johnson, A.H. (1995) Patterns of nutrient loss from unpolluted, old-growth temperate forests: evaluation of biogeochemical theory. *Ecology* **76,** 493–509.

4. Kortelainen, P., Saukkonen, S., and Mattsson, T. (1997) Leaching of nitrogen from forested catchments in Finland. *Glob. Biogeochem. Cy.* **11(4),** 627–638.
5. Campbell, J.L., Hornbeck, J.W., McDowell, W.H., Buso, D.C., Shanley, J.B., and Likens, G.E. (2000) Dissolved organic nitrogen budgets for upland, forested ecosystems in New England. *Biogeochemistry* **49(2),** 123–142.
6. Williams, M.W., Bales, R.C., Brown, A.D., and Melack, J.M. (1995) Fluxes and transformations of nitrogen in a high-elevation catchment, Sierra Nevada. *Biogeochemistry* **28,** 1–31.
7. Coats, R.N. and Goldman, C. (2001) Patterns of nitrogen transport in streams of the Lake Tahoe Basin, California-Nevada. *Water Resour. Res.* **37,** 405–416.
8. Kielland, K. (1994) Amino acid absorption by arctic plants: implications for plant nutrition and nitrogen cycling. *Ecology* **75,** 2373–2383.
9. Nasholm, T., Ekblad, A., Nordin, A., Giesler, R., Hogberg, M., and Hogberg, P. (1998) Boreal forest plants take up organic nitrogen. *Nature* **392,** 914–916.
10. Hunt, A.P., Parry, J.D., and Hamilton-Taylor, J. (2000) Further evidence of elemental composition as an indicator of the bioavailability of humic substances to bacteria. *Limnol. Oceanogr.* **45(1),** 237–241.
11. Currie, W.S., Aber, J.D., McDowell, W.H., Boone, R.D., and Magill, A.H. (1996) Vertical transport of dissolved organic C and N under long-term amendments in pine and hardwood forests. *Biogeochemistry* **35,** 471–505.
12. Neff, J.C., Hobbie, S.E., and Vitousek, P.M. (2000) Nutrient and mineralogical control on dissolved organic C, N, and P fluxes and stoichiometry in Hawaiian soils. *Biogeochemistry* **51,** 283–302.
13. Qualls, R.G. and Haines, B.L. (1991) Geochemistry of dissolved organic nutrients in water percolating through a forest ecosystem. *Soil Sci. Soc. Am. J.* **55,** 1112–1123.
14. Williams, M.W., Hood, E.W., and Caine, N. (2001) The role of organic nitrogen in the nitrogen cycle of a high-elevation catchment, Colorado Front Range, U.S.A. *Water Resour. Res.,* **37,** 2569–2582.
15. McKnight, D.M., Harnish, R., Wershaw, R.L., Baron, J.S., and Schiff, S. (1997) Chemical characteristics of particulate, colloidal and dissolved organic material in Loch Vale Watershed, Rocky Mountain National Park. *Biogeochemistry* **36,** 99–124.
16. Aiken, G.A., McKnight, D.M., Thorn, K.A., and Thurman, E.M. (1992) Isolation of hydrophilic organic acids from water using nonionic macroporous resins. *Org. Geochem.* **18(4),** 567–573.
17. Peterson, B.J., Wollheim, W.M, et al. (2001) Control of nitrogen export from watersheds by headwater streams. *Science* **292,** 86–90.
18. Madole, R.F. (1982) Possible origins of till-like deposits near the summit of the Front Range in north-central Colorado. U.S. Geological Survey Professional Paper 1243. 31 p.
19. Caine, N. (1996) Streamflow patterns in the alpine environment of North Boulder Creek, Colorado Front Range. *Z. Geomorphol. Suppl.* **104,** 27–42.
20. Williams, M.W. and Tonnessen, K.A. (2000) Critical loads for inorganic nitrogen deposition in the Colorado Front Range, U.S.A. *Ecol. Appl.* **10(6),** 1648–1665.
21. Thurman, E.M. and Malcolm, R.L. (1981) Preparative isolation of aquatic humic substances. *Environ. Sci. Technol.* **15,** 463–466.
22. Thurman, E.M. (1985) *Organic Geochemistry of Natural Waters.* Martinus Nijhoff/Dr. W. Junk, Dordrecht, Netherlands. 497 pp.
23. Baron, J., McKnight, D.M., and Denning, A.S. (1991) Sources of dissolved and particulate organic material in Loch Vale Watershed, Rocky Mountain National Park, Colorado, U.S.A. *Biogeochemistry* **15,** 89–109.
24. Caine, N. Personal communication. University of Colorado, Boulder.
25. Huffman, E.W.D. and Stuber, H.A. (1985) Analytical methodology for elemental analysis of humic substances. In *Humic Substances in Soil, Sediment, and Water.* Aiken, G.R., McKnight, D.M., Wershaw, R.L., and MacCarthy, P., Eds. John Wiley & Sons, New York. pp. 433–456.
26. Hornberger, G.M., Bencala, K.E., and McKnight, D.M. (1994) Hydrological controls on dissolved organic carbon during snowmelt in the Snake River near Montezuma, Colorado. *Biogeochemistry* **25,** 147–165.
27. Boyer, E.W., Hornberger, G.M., Bencala, K.E., and McKnight, D.M. (1997) Response characteristics of DOC flushing in an alpine catchment. *Hydrol. Processes* **11,** 1635–1647.
28. Curtis, P.J. and Adams, H.E. (1995) Dissolved organic matter quantity and quality from freshwater and saltwater lakes in east-central Alberta. *Biogeochemistry* **30,** 59–76.
29. Hood, E.W., McKnight, D.M., Williams, M.W. (2001) Sources and chemical quality of dissolved organic carbon (DOC) across an alpine/subalpine ecotone, Green Lakes Valley, Colorado Front Range, U.S.A. *Water Resour. Res.,* in review.
30. Qualls, R.G. and Haines, B.L. (1992) Biodegradability of dissolved organic matter in forest throughfall, soil solution and stream water. *Soil Sci. Soc. Am. J.* **56,** 578–586.
31. McKnight, D.M., Andrews, E.D., Spaulding, S.A., and Aiken, G.R. (1994) Aquatic fulvic acids in algal-rich antarctic ponds. *Limnol. Oceanogr.* **39(8),** 1972–1979.
32. Brooks, P.D., McKnight, D.M., and Bencala, K.E. (1999) The relationship between soil heterotrophic activity, soil dissolved organic carbon (DOC) leachate, and catchment-scale DOC export in headwater catchments. *Water Resour. Res.* **35(6),** 1895–1902.
33. Williams, M.W., Baron, J.S., Caine, N., Sommerfeld, R., and Sanford, Jr., R. (1996) Nitrogen saturation in the Rocky Mountains. *Environ. Sci. Technol.* **30(2),** 640–646.
34. Williams, M.W., Davinroy, T.C., and Brooks, P.W. (1997) Organic and inorganic nitrogen pools in talus fields and subtalus water, Green Lakes Valley, Colorado Front Range. *Hydrol. Processes* **11,** 1747–1760.
35. Sueker, J.K., Ryan, J.N., Kendall, C., and Jarrett, R.D. (2000) Determination of hydrologic pathways during snowmelt for alpine-subalpine basins, Rocky Mountain National Park, Colorado. *Water Resour. Res.* **36(1),** 63–76.
36. Easthouse, K.B., Mulder, J., Christophersen, N., and Seip, H.M. (1992) Dissolved organic carbon fractions in soil and stream water during variable hydrograph conditions at Birkenes, Southern Norway. *Water Resour. Res.* **28(6),** 1585–1596.

| | | |
|---|---|---|
| Received: | July | 23, 2001 |
| Revised: | September | 18, 2001 |
| Accepted: | October | 5, 2001 |
| Published: | October | 30, 2001 |
